# Marine heatwaves in the Northeast Pacific intensify landfalling atmospheric rivers on the west coast of North America

**DOI:** 10.1038/s41598-026-62522-2

**Published:** 2026-07-23

**Authors:** Christoph Renkl, Hyodae Seo, Arthur J. Miller

**Affiliations:** 1 Institute of Geosciences, University of Bonn, Bonn, Germany; 2 Uehiro Center for the Advancement of Oceanography, Department of Oceanography, University of Hawai‘i, Mānoa, Honolulu, Hi USA; 3 Woods Hole Oceanographic Institution, Woods Hole, MA USA; 4 Scripps Institution of Oceanography, University of California, San Diego, La Jolla, CA USA

**Keywords:** Atmospheric rivers, Marine heatwaves, Extreme precipitation, Atmosphere–ocean interactions, Climate sciences, Natural hazards, Ocean sciences

## Abstract

Extreme precipitation along the west coast of North America is often associated with atmospheric rivers (ARs), fueled by evaporation from the ocean. When ARs interact with marine heatwaves (MHWs), they can form compound extreme events with amplified hydrological impacts. Here, we quantify how MHWs influence the intensity and precipitation of landfalling ARs through thermodynamic air–sea interaction processes. We use high-resolution regional coupled ocean–atmosphere ensemble simulations to isolate the influence of large-scale MHW-related sea surface temperature (SST) anomalies while constraining the synoptic-scale atmospheric circulation. Focusing on well-documented AR events during the 2013–16 Northeast Pacific MHW, we show that anomalously warm SSTs enhance evaporation and lower-tropospheric moisture availability, leading to a robust increase in integrated vapor transport and intensified landfalling ARs. The enhanced moisture transport results in earlier onset and substantially increased coastal precipitation, particularly over drought-vulnerable regions of California. Moisture-budget diagnostics demonstrate that this amplification arises from a direct thermodynamic response to SST anomalies, rather than indirect modulation through changes in large-scale atmospheric circulation. Insights gained from this case study identify a thermodynamic pathway linking MHWs and ARs, highlighting the role of persistent oceanic thermal anomalies in shaping compound hydrological extremes under continued climate warming.

**Supplementary Information:**

The online version contains supplementary material available at 10.1038/s41598-026-62522-2.

## Introduction

Hydrological extremes in the eastern North Pacific and along the west coast of North America are strongly shaped by atmospheric rivers (ARs), i.e, elongated, narrow, and transient corridors of enhanced vertically integrated water vapor transport (IVT; see “Methods” section)^1–3^. When ARs encounter lifting mechanisms, such as warm conveyor belts associated with extratropical cyclones or coastal orography, they generate precipitation critical over land for agricultural and water resource management^4–6^. However, ARs can also trigger extreme streamflow^7^, severe flooding^8–12^, and landslides^13^. Landfall characteristics of ARs and their intensity are governed by synoptic and large-scale atmospheric circulation patterns associated with dominant climate modes^14–19^, including the position and strength of mid-latitude cyclones and pressure anomalies over the Northeast Pacific that steer moisture transport and determine the path of anomalous IVT^20,21^.

The intensity of ARs is strongly influenced by ocean–atmosphere coupling through turbulent exchanges of heat and moisture across the air–sea interface^22–27^. These exchanges depend heavily on oceanic thermal boundary conditions and regulate the moisture supply available for transport and precipitation. Upward surface latent and sensible heat fluxes are modified by sea surface temperature (SST) anomalies^24,28,29^, thereby destabilizing the lower atmosphere^27,30^. Persistent, spatially coherent SST anomalies, referred to as marine heatwaves (MHWs), often last weeks to months^31^ and occur frequently in the Northeast Pacific^32–35^. While impacts of MHWs on marine ecology and fisheries are well documented^36–40^, their influence on extreme atmospheric hydrological events remains poorly constrained.

Given that ARs contribute significantly to coastal hazards^41^, understanding how oceanic thermal boundary conditions influence AR intensity is critical for predicting hydrological extremes along the west coast of North America. Previous studies have shown that enhanced evaporation from transient warm SST anomalies can intensify landfalling ARs and heavy precipitation in western North America through increased atmospheric moisture content^25,42^. Warm SST anomalies may also induce a dynamical response in large-scale atmospheric circulation, also altering moisture transport and precipitation patterns along the coast^43^. Currently, it remains unclear whether persistent, basin-scale SST anomalies characteristic of MHWs primarily influence ARs through thermodynamic moistening of the lower troposphere or whether dynamical circulation changes play a comparable role.

**Fig. 1:**
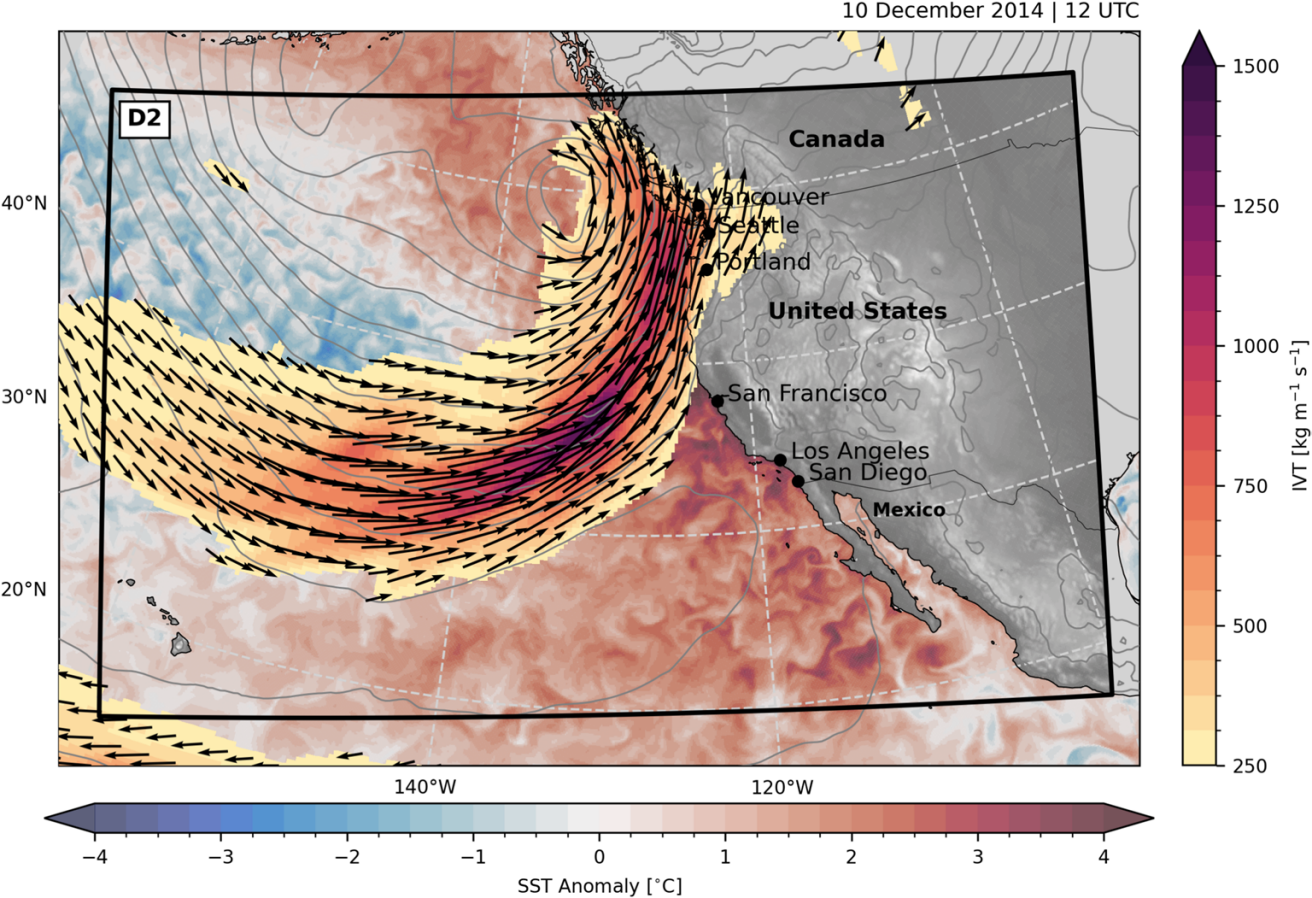
Landfalling atmospheric river (AR) on 10 December 2014. Vertically integrated water vapor transport (IVT, kg m^-1^ s^-1^; vectors at every 4th grid point and shading, only values > 250 kg m^-1^ s^-1^ are shown) and sea level pressure (SLP, hPa; contours in increments of 4 hPa) based on the ERA5 atmospheric reanalysis. Sea surface temperature anomalies (°C; background shading) relative to the 2005–2014 daily climatology based on the GLORYS12v1 ocean reanalysis. The black outline indicates the extent of the coupled model domain D2 (see “Methods” section).

A prominent example of a large-scale, persistent MHW occurred in the Northeast Pacific between 2013 and 2016, commonly referred to as “The Blob”^32,33,44^. This event developed under a strong and persistent high-pressure system over the North American west coast that suppressed wind-driven mixing and wintertime surface cooling, leading to warm SST anomalies across the Gulf of Alaska. Subsequent changes in large-scale atmospheric circulation, including a stronger and southeastward-displaced Aleutian Low^45,46^, shifted the warm SST anomalies eastward to the coast extending southward to Baja California^33^, where they persisted until June 2016^47^. The resulting SST pattern resembled the warm phase of the Pacific Decadal Oscillation (PDO)^48–50^, with pronounced positive SST anomalies along the west coast of North America and negative SST anomaly conditions in the central North Pacific (Fig. 1). The associated atmospheric blocking regime reduced AR activity and contributed to severe drought conditions in California and Oregon^51^. Overall, the large-scale atmospheric state during winter 2014–15 was not conducive to AR landfall along the U.S. West Coast ^21^.

Nevertheless, despite these unfavorable large-scale conditions, several ARs did propagate across and interacted with the MHW before making landfall, including a sequence of events in December 2014. During this period, offshore moisture transport intensified prior to landfall (Fig. 1), producing unusually extreme precipitation exceeding 350mm over 48 hours in parts of northern California, resulting in widespread flooding and wind damage^52^.

From an air–sea interaction perspective, the 2013–16 Northeast Pacific MHW provides a natural laboratory for isolating the impact of persistent, basin-scale SST anomalies on synoptic atmospheric processes. The longevity and spatial coherence of this MHW enabled repeated interactions between multiple ARs and a quasi-stationary warm ocean boundary condition. Observational records and climate projections indicate that both ARs^53–57^ and MHWs^58–62^ are increasing in frequency and amplitude, raising the likelihood that such interactions of compound high-impact event will occur more often. Hence, the 2013–16 Northeast Pacific MHW event enables an explicit assessment of how sustained diabatic heating and enhanced evaporation from anomalously warm SSTs influence AR intensity and downstream precipitation.

In this study, we investigate how persistent MHWs influence the intensity and hydrological impacts of landfalling ARs through thermodynamic air–sea interactions. Using multi-ensemble, high-resolution regional coupled ocean–atmosphere simulations, we conduct controlled experiments designed to isolate the large-scale SST anomaly pattern associated with the Northeast Pacific MHW while constraining synoptic-scale atmospheric circulation (see Methods). This framework allows us to separate thermodynamic effects arising from enhanced surface evaporation and moistening of the lower troposphere from a potential dynamical response of the atmospheric circulation. Our results show that persistent warm SST anomalies promote enhanced evaporation, thus acting as a sustained moisture source that amplifies AR intensity during oceanic transit, leading to earlier onset and enhanced precipitation upon landfall. These findings suggest a thermodynamic pathway linking MHWs and ARs, with important implications for compound ocean–atmosphere extremes in a warming climate.

## Results

### MHWs intensify ARs and increase coastal precipitation

**Fig. 2:**
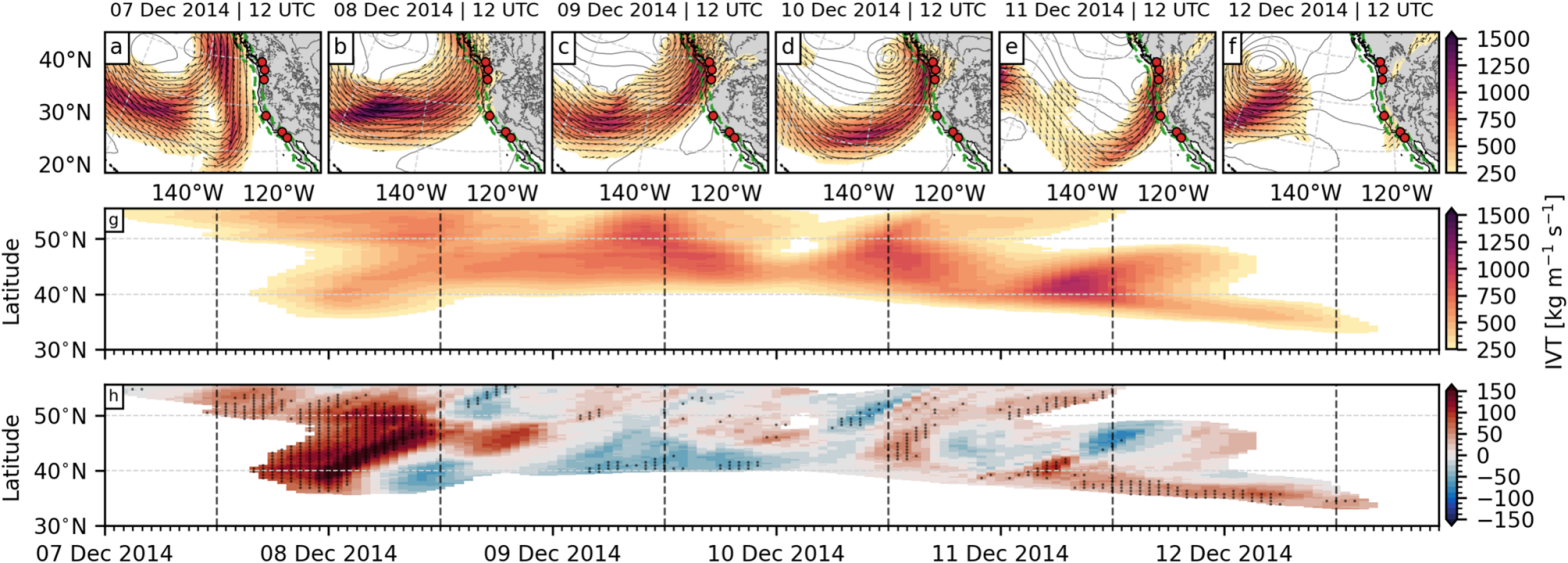
Landfalling atmospheric rivers (ARs) during the period 7–12 December 2014 predicted by the coupled model. (**a**)–(**f**) Daily snapshots of ensemble mean vertically integrated water vapor transport (IVT, kg m^-1^ s^-1^; vectors at every 20th model grid point and shading, only values > 250 kg m^-1^ s^-1^ are shown) and sea level pressure (SLP, hPa; contours in increments of 5 hPa) based on the MHW simulation. Green dashed lines delineate the area ± 50 km from the coast and red dots mark major cities. (**g**) Hovmöller diagram of ensemble mean IVT (MHW simulation) zonally averaged over the area ± 50 km from the coast. (**h**) Hovmöller diagram of differences in ensemble mean IVT (MHW − noMHW) zonally averaged over the area ± 50 km from the coast. Statistically significant differences (two-sided Student’s *t*-test, *p* ≤ 0.01) are marked by gray dots every 8 grid points in the latitudinal direction for visual clarity. Values are only shown when IVT magnitude > 250 kg m^-1^ s^-1^ in either the MHW or noMHW simulation. Vertical dashed lines indicate the time at which the snapshots in panels a–f are taken.

**Fig. 3:**
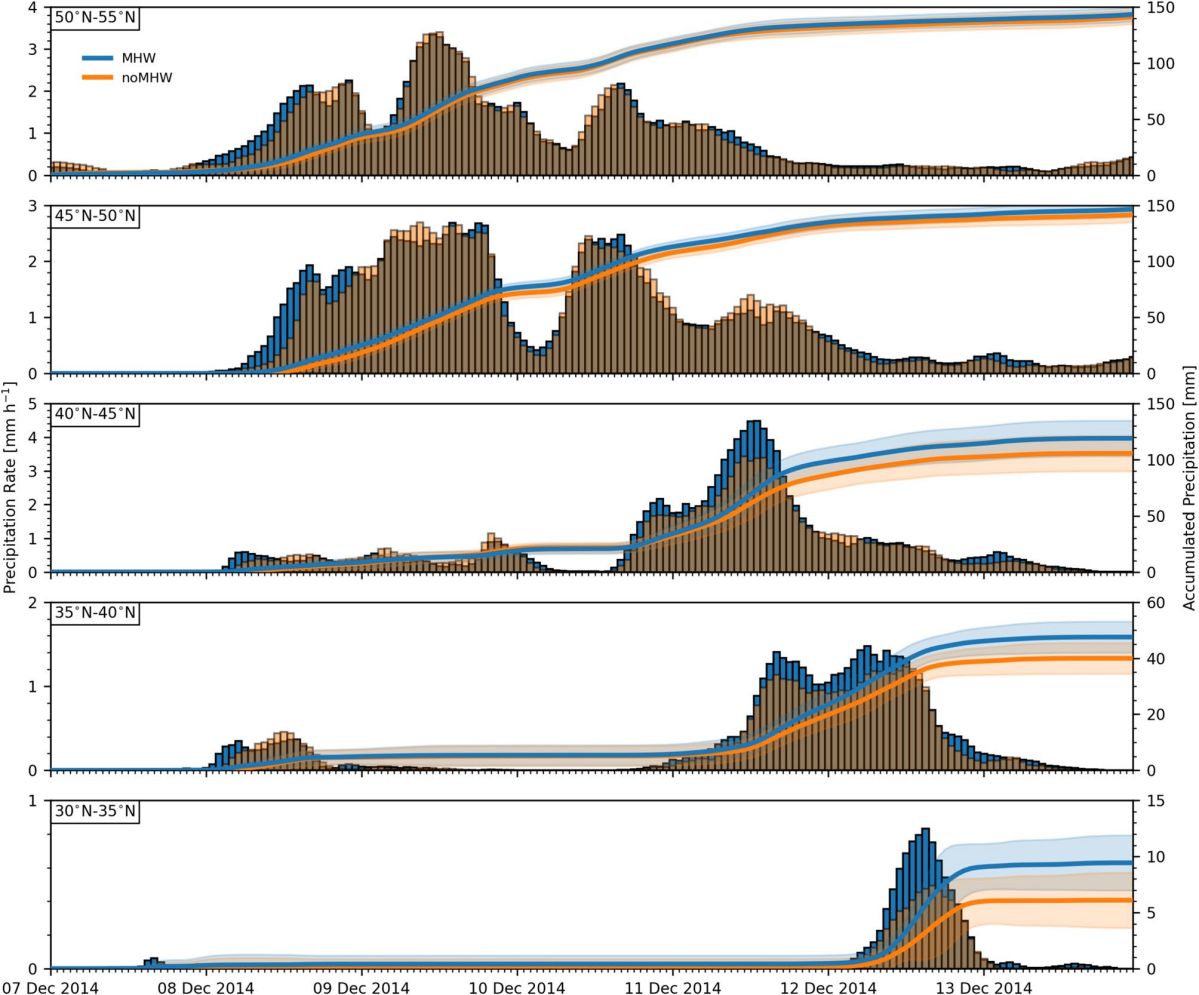
Precipitation along the west coast of North America during AR events during the period 7–13 December 2014 predicted by the coupled model. Ensemble mean hourly precipitation rate (mm h^-1^; bars, left axis) and accumulated precipitation (mm; lines, right axis) averaged over a 100 km inland area along the coast in five latitude bands from the MHW (blue) and noMHW (orange) simulations. Shading around the lines denote ± 1 standard deviation of the differences between the individual MWH and noMHW ensemble simulations around their mean. Please note the different scales along the *y*-axis in each panel.

During the period 7–13 December 2014, a sequence of ARs, defined by IVT magnitude exceeding 250 kg m^-1^ s^-1^ (see Methods), interacted with warm SST anomalies associated with the 2013–16 Northeast Pacific MHW and made landfall along the west coast of North America (Fig. 2a–f). These events were associated with enhanced integrated water vapor (IWV; Fig. S4) along the coast and induced strong precipitation over inland (Fig. 3), temporarily alleviating exceptional drought conditions in northern California, but also causing widespread flooding^52^. The MHW simulation of coupled model, representing realistic conditions consistent with observation including the large-scale SST anomalies, captures the atmospheric circulation during this period as well as the structure, timing, and intensity of the ARs (Fig. S3).

Persistent AR conditions were maintained by upstream development of mesoscale frontal waves associated with multiple IVT “cusps”^23,52^, that are well captured by the model. The Hovmöller diagram in Fig. 2g shows the IVT magnitude zonally averaged over a 100 km coastal strip as a function of latitude and time (only values > 250 kg m^-1^ s^-1^ are shown). A sequence of ARs, associated with pulses of elevated IVT, made landfall along the coast of British Columbia, Washington, and Oregon before propagating southward to California. Moderate AR conditions, with maximum IVT > 500 kg m^-1^ s^-1^, persisted along much of the coast for approximately 99 hours. Peak IVT exceeded 1000 kg m^-1^ s^-1^ for several hours on 11 December 2014, classifying this episode as an AR Category 3 event^63^.

To test the impact of the contemporary MHW on these AR events, we performed a sensitivity experiment where large-scale temperature anomalies have been removed in the ocean model, henceforth referred to as noMHW simulation (see Methods). Differences between the simulations (MHW − noMHW) indicate that AR intensity during the study period was enhanced by the presence of warm SST anomalies associated with the MHW (Fig. 2h). Depending on time and location, statistically significant increases in IVT, zonally averaged over the coastal band, reach 11–210 kg m^-1^ s^-1^, corresponding to an intensification of landfalling ARs by 3–56 % relative to the noMHW simulation (two-sided Student’s *t*-test; *p* ≤ 0.01). While the spatial extent of AR conditions (IVT > 250 kg m^-1^ s^-1^) remains largely unchanged, small shifts in the position of the AR core lead to localized reductions in coastal IVT in some instances.

In the noMHW simulation, the maximum IVT along the coast exceeds the extreme intensity threshold (1000 kg m^-1^ s^-1^) only once (not shown), effectively downgrading the episode to an AR category 2 event ^63^. The total duration of AR conditions is comparable between the simulations, with 136 hours and 134 hours for the MHW and noMHW cases, respectively. This suggests a primarily thermodynamic response to the large-scale SST anomalies that is further explored below.

Figure 3 shows ensemble-mean hourly precipitation rates spatially averaged over a 100 km inshore region and 5° latitude bins, along with accumulated precipitation for the period 7–13 December 2014, averaged over the same region, in both simulations. In the presence of the MHW (blue), precipitation begins several hours earlier and exhibits higher peak rates relative to the noMHW simulation (orange). Consequently, accumulated precipitation in the MHW simulation increases over the one-week period, although the magnitude of this increase varies regionally. Along the northern west coast (British Columbia and Washington; 45–55° N), the accumulated precipitation averaged over the inshore region exceeds 140 mm in both simulations. In these regions, the MHW simulation predicts a marginal increase in total precipitation of 2 % and 4 %, respectively, relative to the noMHW simulation. In contrast, coastal California and northern Baja California (30–44° N), regions often affected by droughts, receive less precipitation during that one-week period (9-119 mm in the MHW simulation). However, the increases of 19–55 % relative to the noMHW simulation are substantially larger than in the northern part of the model domain.

### Persistent warm SST anomalies enhance evaporation and generate a moisture anomaly

**Fig. 4:**
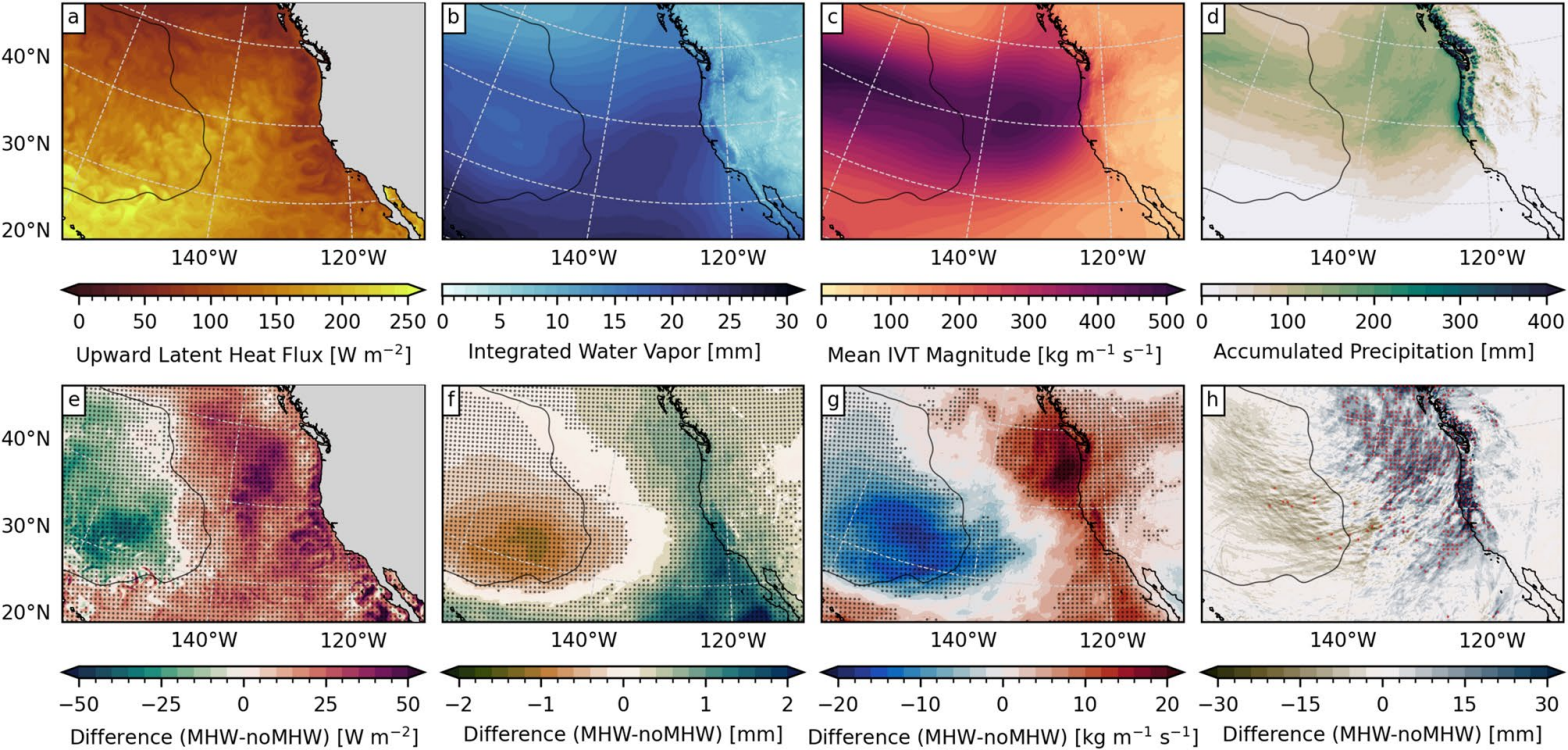
Ensemble time mean fields of the MHW simulation (top) and differences (MHW − noMHW; bottom) for the period 7–20 December 2014. (**a**,** e**) Upward latent heat flux (W m^-2^), (**b**, **f**) vertically integrated water vapor (mm), (**c**, **g**) mean of IVT magnitude (kg m^-1^ s^-1^), and (**d**, **h**) accumulated precipitation (mm) from MHW simulation. The black contours mark the zero line illustrating the large-scale SST anomalies in the MHW simulation. Stippling indicates statistically significant differences (two-sided Student’s *t*-test, *p* ≤ 0.01).

During winter, the surface ocean in the Northeast Pacific loses heat to the atmosphere primarily through turbulent heat exchange, dominated by upward latent heat flux associated with evaporation. This is captured by the MHW simulation, showing latent heat flux is strongest northeast of Hawai‘i, and weaker along the coast and in the northern part of the domain (Fig. 4a). Notably, the regions of enhanced heat loss near 140° W coincides with the weakest warm SST anomaly during the MHW^47^. While this pattern broadly resembles long-term climatological conditions^64^, the relatively short analysis period is strongly dominated by repeated AR events. This shapes the mean conditions in the model.

Evaporation from the ocean provides a primary moisture source to the atmosphere, resulting in a corresponding spatial distribution of IWV, with a slight eastward displacement, presumably due to advection by the mean flow (Fig. 4b). Over the ocean, IWV exhibits a pronounced northwest–southeast gradient, with relatively dry air in the northwest and maximum moisture content (IWV > 30 mm) in the southern part of the domain. North of Hawai‘i, where SST anomalies are negative, a local moisture minimum is evident.

The influence of AR activity during the simulation period is reflected in a zonal corridor of enhanced mean IVT magnitude near 40° N (Fig. 4c), accompanied by elevated accumulated precipitation along this corridor, indicating moisture recycling within the ARs (Fig. 4d). Precipitation maxima occur near the coast, where moisture-laden air is lifted by orography, leading to condensation and enhanced precipitation. As a result, less moisture penetrates inland, yielding lower IWV and IVT values over the continent (Fig. 4b–c).

To illustrate the influence of large-scale SST anomalies on these mean conditions, Fig. 4e–h show the ensemble time mean difference between the model simulations (MHW − noMHW). Positive SST anomalies associated with the MHW lead to enhanced latent heat fluxes and increased evaporation compared to the noMHW simulation (Fig. 4e). Warmer SSTs raise near-surface saturation vapor pressure, strengthening vertical humidity gradients at the air–sea interface, and promoting evaporation (Fig. S5). Differences in latent heat flux reach up to 50 W m^-2^, corresponding to increases of up to 60 % relative to the noMHW simulation. The resulting anomalous moisture source leads to increased precipitable water (IWV), particularly near the coast (Fig. 4f). While vertical redistribution of moisture reduces the relative increase in IWV, values still rise by up to 10 % relative to the noMHW simulation. As ARs advect this additional moisture eastward, mean IVT increases by 10–20 kg m^-1^ s^-1^ along the coast, contributing to enhanced coastal precipitation (Fig. 4g–h). This increase in mean IVT of 5–10 % with respect to the noMHW simulation illustrates clearly the role of the MHW in intensifying individual ARs during the time period (Fig. 2).

Since air masses within much of the ARs are nearly saturated, the maximum latent heat flux is found upstream of the AR core, where strong winds and large humidity gradients are present behind the cold front^65,66^. Horizontal advection relative to cyclone propagation leads to moisture accumulation along frontal boundaries, sustaining high water vapor content within the AR^67^. This process is further examined in the following section.

### MHWs intensify ARs primarily through thermodynamic processes

**Fig. 5:**
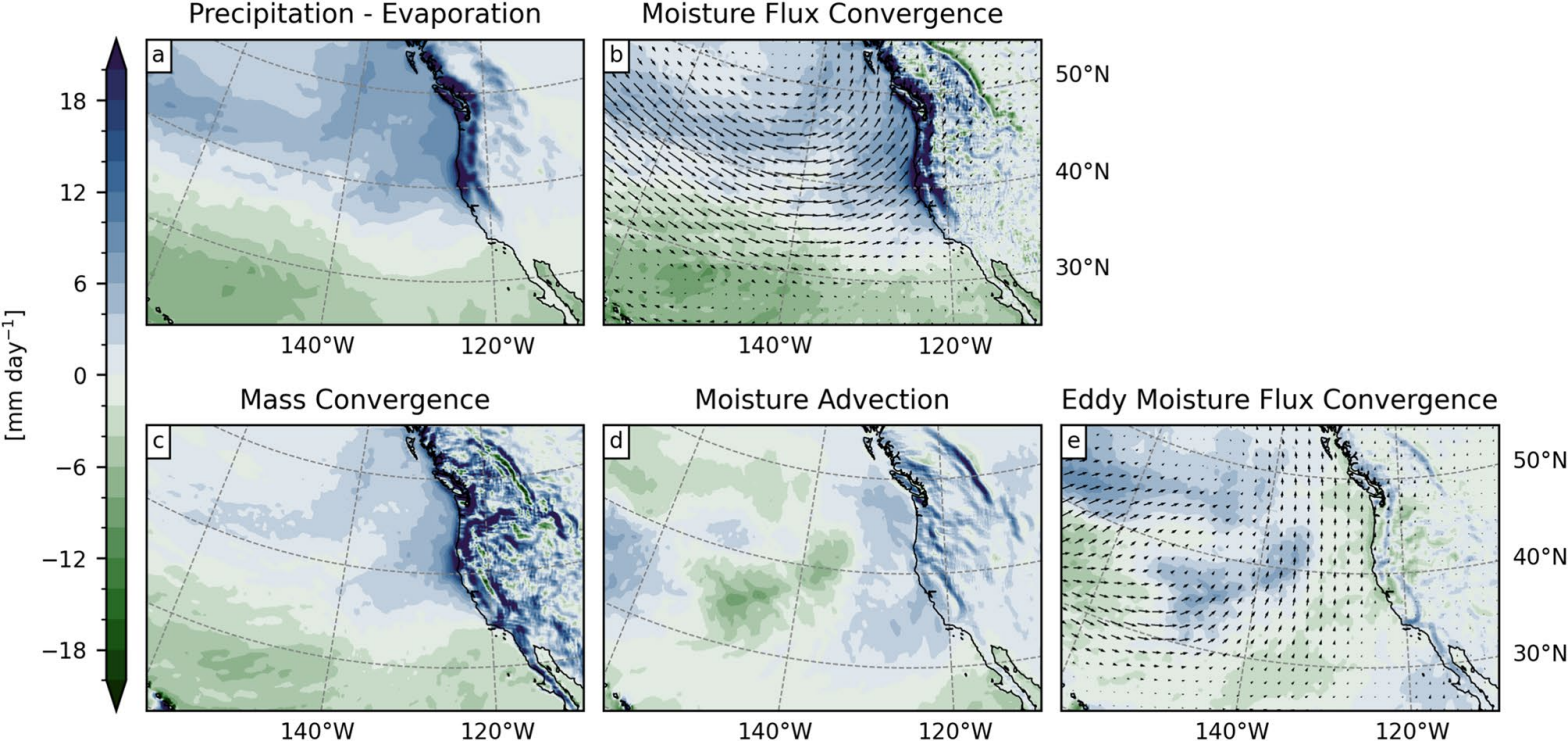
Ensemble mean time-average moisture budget for the period 7–20 December 2014 predicted by the coupled MHW simulation. (**a**) Net surface moisture flux defined as precipitation minus evaporation and (**b**) moisture flux convergence. Contribution to moisture flux convergence by (**c**) moisture-weighted mass convergence, (**d**) advection by mean flow, and (**e**) transient eddy moisture flux. All fields have been smoothed with a 9 × 9 grid point centered rolling mean. Vectors in panels (b) and (e) show the mean IVT and mean eddy moisture flux, respectively.

To isolate the physical mechanisms responsible for AR intensification, we evaluate the vertically integrated moisture budget, relating the net surface moisture flux (precipitation minus evaporation) to moisture flux convergence (see Methods). Over the analysis period, mean conditions predicted by the MHW simulation exhibit a dipole structure, with net precipitation associated with moisture flux convergence in the northern half of the domain and net evaporation linked to moisture flux divergence over the ocean in the southern part (Fig. 5a–b). Maximum precipitation occurs along the coast, collocated with strongest moisture flux convergence resulting from interaction of the flow with steep topography. Again, given the short analysis period and frequent synoptic disturbances, the mean state is biased toward conditions with relatively strong winds and moisture transport.

Moisture flux convergence is decomposed into contributions from horizontal advection by the mean flow across moisture gradients, moisture-weighted mass convergence, and convergence of transient eddy moisture flux^68–71^. The contribution by horizontal mass convergence is related to vertical motion. Over the southern part of the domain, negative values (moisture flux divergence) reflect large-scale subsidence associated with the Hadley circulation, suppressing precipitation and sustaining net evaporation (Fig. 5c). At mid-latitudes, eddy-driven ascent contributes to moisture convergence and precipitation along the storm track. Upon reaching the mountainous coastline, interaction with topography forces further convergence and orographic lift, producing a pronounced coastal precipitation maximum. Cyclonic mean flow over the Gulf of Alaska advects drier air southward over the ocean while transporting moisture from lower latitudes toward the coast (Fig. 5d). As ARs propagate across the Northeast Pacific, mean eddy moisture flux convergence further enhances precipitation along the storm track (Fig. 5e).

**Fig. 6:**
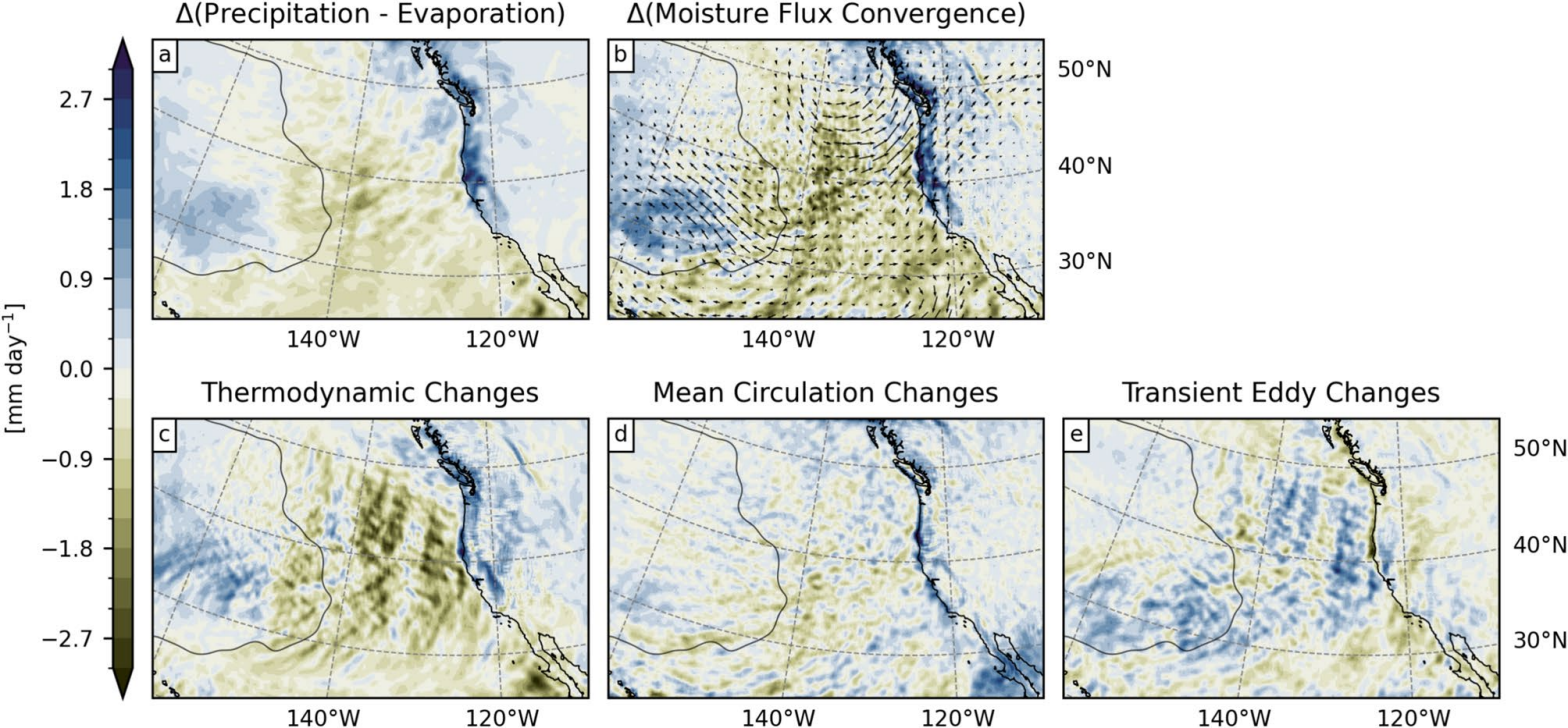
Changes in time-averaged ensemble mean moisture budget with respect to the noMHW simulation. Differences (MHW − noMHW) in (**a**) net surface moisture flux and (**b**) moisture flux convergence. Contribution to changes in mean moisture budget by (**c**) thermodynamics, (**d**) mean circulation, and (**e**) transient eddy moisture flux. All fields have been smoothed with a 9 × 9 grid point centered rolling mean. Differences in IVT are shown by vectors in panel (b) that arise through both thermodynamic and dynamic changes.

Figure 6 shows the differences in the vertically integrated moisture budget between the model simulations (MHW − noMHW), illustrating both thermodynamic and dynamic changes. In the presence of MHW-related SST anomalies, evaporation from the warmer ocean surface increases, creating an anomalous moisture source and enhanced divergence of the mean moisture flux over the ocean (Fig. 6a–b). Concurrently, precipitation and moisture flux convergence increase along the coast. These differences between the MHW and noMHW simulations arise from changes in thermodynamics, mean circulation, and transient eddies. Analysis of a linearized formulation of the bulk formula for latent heat flux reveals that the increased evaporation is almost entirely driven by thermodynamic changes due to an enhanced vertical moisture gradient at the air–sea interface (Fig. S5). This is corroborated by the more rigorous decomposition by Bartusek et al.^24^ who showed a robust influence of SST anomalies on latent heat flux variations under ARs on interannual timescales.

Following a widely used methodology^69^, changes in moisture flux convergence are decomposed into contributions from thermodynamic effects, mean circulation, and transient eddy moisture flux (see Methods). The thermodynamic contribution, driven by increased specific humidity under MHW conditions (Fig. 4b, f), exhibits a spatial pattern closely resembling the total moisture flux convergence anomaly, with enhanced moisture export over the MHW region and increased convergence along the coast (Fig. 6c). In contrast, circulation-driven changes are weaker and less spatially coherent (Fig. 6d). Transient eddy moisture fluxes partially offset the thermodynamic contribution over the ocean, consistent with enhanced moisture recycling, but do not fully compensate for the anomalous evaporation (Fig. 6e).

These results demonstrate that the intensification of ARs during December 2014 (Fig. 2) and the associated increase in precipitation (Fig. 3) arise primarily from thermodynamic processes associated with persistent MHW conditions, rather than substantial changes in synoptic-scale circulation. Enhanced evaporation due to anomalously warm SSTs generates a robust moisture anomaly over the Northeast Pacific, that is subsequently advected by ARs toward the coast, increasing IVT and precipitation upon landfall. While dynamical adjustments and transient eddy processes contribute to the regional moisture budget, their influence is secondary to the thermodynamic moistening driven by the oceanic boundary condition. However, it is important to note that this does not render the circulation unimportant as it provides a mechanism for the additional moisture to be transported toward the coast.

## Discussion

In this study, we examined the multiscale air–sea interactions that occur when transient ARs propagate across persistent warm SST anomalies associated with large-scale MHWs in the Northeast Pacific. Our results show that anomalously warm surface waters enhance upward latent heat flux and evaporation, increasing lower-tropospheric moisture availability during AR transit (Fig. 4). This additional moisture is picked up by ARs, augmenting IVT and amplifying AR intensity prior to landfall. Our simulations indicate that the presence of the 2013–16 Northeast Pacific MHW intensified landfalling ARs by up to 56 % relative to the noMHW simulation, effectively upgrading their classification to an AR Category 3 event in this case^63^. Upon landfall, enhanced moisture transport results in stronger precipitation, with increases of 19–55 % with respect to the noMHW simulation over coastal California (Fig. 3), a region that was experiencing severe drought conditions at the time ^72^.

Analysis of the vertically integrated moisture budget indicates that this response is dominated by thermodynamic processes that amplify moisture transport steered by the large-scale circulation. Enhanced evaporation over the MHW region provides anomalous moisture that is exported downstream toward the coast, while transient eddy moisture flux convergence partially offsets this signal over the ocean (Fig. 6). The net effect is increased moisture flux convergence along the coast, where orographic lifting further amplifies precipitation. These results suggest a thermodynamic pathway through which persistent oceanic thermal anomalies intensify ARs embedded in the atmospheric circulation and increase coastal precipitation, thereby elevating the risk of flooding, landslides, and extreme streamflow.

Our findings are consistent with previous observational and modeling studies linking increases in AR frequency and intensity to enhanced atmospheric moisture content under warming conditions, as expected from the Clausius–Clapeyron relationship^53–57^. Positive SST anomalies have been identified as a key contributor to this thermodynamic response ^73^. While our study focused on a particular event, the projected increase in MHW frequency, intensity, and duration under continued climate warming^59–62^ implies a growing potential for MHWs to precondition the atmosphere for more intense AR events, increasing the likelihood of compound ocean–atmosphere extremes.

On longer time scales, diabatic heating associated with persistent SST anomalies may also influence large-scale circulation patterns and further amplify hydrological impacts^43,57^. At the same time, the atmospheric circulation can also act to reduce SST anomalies^74^. In the present study, however, large-scale atmospheric circulation is constrained by boundary conditions and the relatively short analysis period, limiting the emergence of pronounced dynamical adjustments.

Our modeling framework effectively enables isolation of the thermodynamic influence of SST anomalies on individual AR events over synoptic time scales, beyond which deterministic AR prediction skill declines rapidly^75–77^. This contrasts with prior work that has emphasized dynamical processes and circulation anomalies as primary drivers of atmospheric river variability on seasonal to longer time-scales^43^.

The spatial structure of the atmospheric response closely reflects the underlying SST anomaly pattern. Regions of anomalously warm SSTs exhibit enhanced latent heat fluxes and increased moisture supply to the atmosphere, whereas areas with cooler SST anomalies show an opposing response. This spatial correspondence suggests the importance of accurately characterizing the distribution, intensity, and evolution of SST anomalies for understanding how MHWs modulate AR behavior. We note, however, that SST anomalies were defined relative to the 2005–2014 climatology, which may itself be biased toward warmer conditions. A longer climatological baseline would likely increase the magnitude of the warm anomalies and reduce cold anomalies, suggesting that the AR intensification diagnosed here represents a conservative estimate of the MHW impact.

Beyond the direct downstream precipitation response, enhanced air–sea fluxes associated with MHWs may also influence the evolution of upstream cyclones and sequences of successive AR events. Strong winds and cold, dry air in the post-frontal sector promote large latent and sensible heat fluxes due to increased vapor pressure deficits and air–sea temperature contrasts^66,67,78,79^. Enhanced evaporation in these regions can sustain moisture convergence ahead of the cold front and contribute moisture to the warm conveyor belt of upstream cyclones, potentially preconditioning subsequent ARs^67,79,80^. While this process may contribute to the enhanced IVT observed during parts of the December 2014 AR sequence, a detailed assessment of cyclone–AR feedback is beyond the scope of this study.

Finally, while this study focuses on the atmospheric responses to MHW-related SST anomalies, ARs also exert substantial influences on the ocean surface. AR-related wind forcing is known to induce rapid SST cooling^81^ and drive Ekman transport toward the coast, increasing coastal sea level and alongshore current ^65,66^, and contribute to advective warming ahead of the AR in some coastal regions^82^. In addition, intense precipitation associated with ARs can modify upper ocean salinity and stratification along the coast^66,83,84^. These oceanic responses may partially offset or reinforce MHW conditions, introducing additional feedbacks within the coupled ocean–atmosphere system that shape the overall response in ARs by influencing air-sea fluxes. While these processes are captured in the coupled model, quantifying these two-way interactions was beyond the scope of the present study and remains an important open question^74^. Future studies combining longer simulations, expanded ensemble strategies, and targeted ocean diagnostics will be required to fully resolve the coupled evolution of MHWs, ARs, and the coastal ocean.

## Methods

### Ocean-atmosphere coupled model

This study is based on ensemble simulations conducted with the Scripps Coupled Ocean–Atmosphere Regional (SCOAR) modeling system^85–89^. SCOAR couples the Weather Research and Forecasting Model (WRF, version 4.2.2)^90^ with the Regional Ocean Modeling System (ROMS, version 3.8)^91^, enabling interactive exchanges of momentum, heat, and moisture across the air–sea interface. Air–sea fluxes are computed using version 3.5 of the COARE bulk flux parameterization ^92–94^ implemented within the WRF surface layer scheme.

The ensemble design was chosen to isolate the thermodynamic response to SST anomalies from intrinsic atmospheric variability while preserving the same synoptic evolution imposed by boundary forcing. The 11 ensemble members, generated through small perturbations to atmospheric initial conditions staggered by ± 1 – 5 hours across 00 UTC on 1 December 2014, provide a robust estimate of the forced signal at synoptic time scales, as evidenced by the consistency and statistical significance of the ensemble-mean responses.

We employ a one-way nested atmospheric configuration over the Northeast Pacific and western North America (Fig. S1). The outer atmosphere-only domain (D1; 30km horizontal grid spacing) is forced by hourly fields of the fifth generation ECMWF atmospheric reanalysis (ERA5)^95^ that are prescribed at the lateral boundaries of D1. Over this domain, WRF is actively integrated to provide physically consistent boundary conditions to the inner domain (D2; 7.5km horizontal grid spacing) that is fully coupled to the ocean model. The purpose of this approach is to avoid inconsistencies and minimize errors that could arise from directly prescribing regridded ERA5 data along the lateral boundaries of D2.

To maintain consistency with the observed large-scale circulation, spectral nudging is applied only in D1 above the planetary boundary layer for temperature, specific humidity, wind, and geopotential height on zonal and meridional length scales exceeding 5745 km and 3360 km, respectively (wave number 3 in *x* and *y* direction)^96^. No nudging is applied within the coupled domain (D2), allowing the atmosphere and mesoscale air–sea interactions to evolve freely. Both atmospheric domains share 33 vertical levels extending to 50 hPa and use the same parameterizations. Details about the applied WRF model physics are summarized in the Supplementary Information (Tab. S1).

Within the coupled domain D2, WRF and ROMS share the same horizontal grid and land–sea mask. The ocean component uses 30 terrain-following *s*-coordinate vertical levels^97^, with stretching parameters *θ*_s_ = 7, *θ*_b_ = 2, and *h*_c_ = 300 m. Open boundary conditions for temperature, salinity, sea surface height, and currents are prescribed from daily GLORYS12v1 ocean reanalysis^98^. Tidal forcing is imposed using the Oregon State University TPXO 1/12° regional tidal solution for the Pacific Ocean (PO 2009)^99^, and river discharge is prescribed from the Global River Flow and Continental Discharge Dataset^100,101^. Further details about the numerical schemes and parameterizations used are summarized in the Supplementary Information (Tab. S1).

### Model experiments

The experimental design is structured to isolate the thermodynamic influence of large-scale persistent MHW SST anomalies while preserving realistic synoptic atmospheric forcing and local air–sea coupling.

We first conducted a 10-year ocean-only hindcast simulation over D2 (CLIM; Table 1) for 2005–2014, forced by hourly ERA5 fields of sea level pressure, air temperature and specific humidity at 2 m, 10 m wind, precipitation, net shortwave and downward longwave radiation. These forcing fields are converted internally into air–sea fluxes of momentum, heat, and moisture consistent with the simulated surface ocean using COARE implemented in ROMS.

Ocean initial and boundary conditions were constructed from the daily GLORYS12v1 reanalysis ^98^. The role of this ocean-only hindcast simulation is to i) evaluate and tune ocean model physics to identify the optimal configuration for the ocean model (see Table S1 for details), ii) construct a daily ocean temperature climatology used to define MHW anomalies, and iii) provide initial conditions for the coupled spin-up simulation (SPIN-UP; Table 1).

Following CLIM, the one-month coupled spin-up simulation (SPIN-UP) is then conducted for November 2014 to allow for the ocean and atmosphere to adjust dynamically prior to the main analysis period. From the final state of SPIN-UP, two ensembles of coupled simulations are launched for December 2014, one retaining the realistic marine heatwave SST anomaly (MHW; Table 1), consistent with observations, and one with the anomaly removed from the initial conditions as described below (noMHW; Table 1). In both simulations, the ocean—including the SST—evolves freely and interacts with the atmospheric component of SCOAR, thereby fully capturing air–sea coupling processes and potential feedback mechanisms.

The large-scale SST anomaly associated with the marine heatwave is isolated by first subtracting the daily climatological mean based on CLIM from the SPIN-UP ocean state at 00 UTC on 1 December 2014 (Fig. S2a–b). Next, the full anomaly field is low-pass filtered using a locally estimated scatterplot smoothing (LOESS) smoother^102–104^, with half-width (effective cutoff) length scale of 5° (210–320 km) ^105^, applied to anomalies on each model level. The basin-scale SST anomaly closely resembles the warm phase of the Pacific Decadal Oscillation (PDO)^48–50^, with warm anomalies along the west coast of North America and cooler anomalies in the central North Pacific (Fig. 1, Fig. S2c). This filtered basin-scale SST anomaly (Fig. S2c) constitutes the marine heatwave that is removed from the initial conditions in the noMHW simulation (Fig. S2d). The same anomaly removal is also applied to the ocean boundary conditions. Subtracting only the large-scale SST pattern associated with the 2013–16 MHW preserves mesoscale ocean variability in the noMHW simulation that would otherwise spin up due to the interactive coupling with the atmosphere. This approach is comparable to the atmosphere-only experiments by Bischof et al.^106^.

**Table 1:** Summary of model experiments using the SCOAR Modeling System.

Experiment	Forcing	Period	MHW	Ensemble Size
CLIM	ERA5	2005–2014	Yes	1
SPIN-UP	WRF (coupled)	1 Nov.–1 Dec. 2014	Yes	1
MHW	WRF (coupled)	1–20 December 2014	Yes	11
noMHW	WRF (coupled)	1–20 December 2014	No	11

### Atmospheric river identification

Atmospheric river (AR) intensity is quantified using integrated vapor transport (IVT), defined as the mass-weighted vertical integral of the horizontal moisture flux^63^:

1 $$\begin{aligned} \text {IVT} = - \frac{1}{g} \int _\text {1000 hPa}^\text {300 hPa} q \boldsymbol{u} \, dp, \end{aligned}$$

where ">*u* is the horizontal wind vector, *q* is specific humidity, and *g* is acceleration due to gravity.

Equation (1) is used to calculate IVT from hourly WRF output on eight pressure levels at 1000, 925, 850, 700, 600; 500, 400, and 300 hPa. Levels below the surface (e.g., in mountainous regions) are masked following Seager and Henderson^70^. AR conditions are defined when IVT magnitude exceeds 250 kg m^-1^ s^-1^, a commonly used threshold in mid-latitudes AR studies. We do not impose additional geometric or duration constraints^107^, which are difficult to apply consistently within a limited-area regional domain.

### Moisture budget diagnostics

To diagnose the mechanisms linking SST anomalies to precipitation changes, we evaluate the vertically integrated atmospheric moisture budget. For time-averaged conditions, the steady-state atmospheric water vapor budget can be written as^68–70^,

2 $$\begin{aligned} \begin{aligned} \overline{P - E}&= - \frac{1}{g \rho _\text {w}} \nabla \cdot \int _{0}^{p_\text {s}} \overline{q \boldsymbol{u}} \, dp \\&= - \frac{1}{g \rho _\text {w}} \int _{0}^{p_\text {s}} \left[ \underbrace{\overline{\boldsymbol{u}} \cdot \nabla \overline{q}}_\text {(A)} + \underbrace{\overline{q} (\nabla \cdot \overline{\boldsymbol{u}})}_\text {(B)} + \underbrace{\nabla \cdot (\overline{q' \boldsymbol{u}'})}_\text {(C)} \right] \, dp \underbrace{- \frac{1}{g \rho _\text {w}} \overline{q_\text {s} \boldsymbol{u}_\text {s}} \cdot \nabla p_\text {s}}_\text {(D)}, \end{aligned} \end{aligned}$$

where *P* is precipitation, *E* is evaporation, *ρ*_w_ is the density of water, and the overline denotes the time average.

The term on the right-hand side of Eq. (2) is the mean moisture flux convergence, that can be decomposed into contributions from mean horizontal advection (A), moisture-weighted mass convergence (B), and transient eddy moisture flux convergence (C). As part of this decomposition, a correction term (D) involving surface values (indicated by a subscript ‘s’) arises from moving the divergence operator under the integral. Since this term is only significant in regions with strong changes in topography and often ignored^70^, we do not discuss it in our moisture budget analysis.

To attribute differences in $\overline{P - E}$ between the MHW and noMHW simulations to changes in moisture content versus circulation, we decompose the MHW − noMHW difference in moisture flux convergence into contributions from thermodynamic changes (A), mean circulation changes (B), and transient eddy moisture fluxes (C)^69^:

3 $$\begin{aligned} \Delta (\overline{P - E}) \approx \underbrace{- \frac{1}{g \rho _\text {w}} \int _{0}^{p_\text {s}} \nabla \cdot \left[ (\Delta \overline{q}) \overline{\boldsymbol{u}}_\text {noMHW} \right] \, dp}_\text {A} \underbrace{- \frac{1}{g \rho _\text {w}} \int _{0}^{p_\text {s}} \nabla \cdot \left[ \overline{q}_\text {noMHW} (\Delta \overline{\boldsymbol{u}})\right] \, dp}_\text {B} \underbrace{- \frac{1}{g \rho _\text {w}} \int _{0}^{p_\text {s}} \nabla \cdot \Delta (\overline{q' \boldsymbol{u}'}) \, dp}_\text {C} \end{aligned}$$

where

4 $$\begin{aligned} \Delta ( \cdot ) = ( \cdot )_\text {MHW} - (\cdot )_\text {noMHW} \end{aligned}$$

denotes the difference between the MHW and noMHW simulations. Note that the differences in the surface correction term in Eq. (2) are omitted.

We evaluate Eqs. (2) and (3) using hourly WRF output on 10 pressure levels at 1000, 925, 850, 700, 600, 500, 400, 300, 200, and 100 hPa. Following Seager and Henderson^70^, values of *u* and *q* from the first pressure level above *p*_s_ are used to represent the layer below and the surface. Time-mean quantities are computed over the period 7–20 December 2014, and transient eddy fluxes are calculated from hourly anomalies relative to this mean, thereby capturing moisture transport by transient mid-latitude disturbances^70^.

## Supplementary Information

Below is the link to the electronic supplementary material.

Supplementary Material 1

## Data Availability

All data required to configure and run the SCOAR modeling system are publicly available: ERA5 data are made available by Copernicus Climate Change Service (https://cds.climate.copernicus.eu), GLORYS12v1 by Copernicus Marine Environment Monitoring Service (10.48670/moi-00021), TPXO regional tidal solution for the Pacific Ocean by Oregon State University (https://www.tpxo.net/regional), and Global River Flow and Continental Discharge Dataset by the NSF National Center for Atmospheric Research (10.5065/D6V69H1T).

## Code availability

All source code of the SCOAR modeling system is accessible on GitHub: ROMS source code (https://github.com/myroms/roms), WRF source code (https://github.com/wrf-model/WRF), WRF preprocessing system (WPS, https://github.com/wrf-model/WPS), and SCOAR source code, including tutorials (https://github.com/SCOAR-model).
