# Supplementary material for: Marine heatwaves in the Northeast Pacific intensify landfalling atmospheric rivers on the west coast of North America
Source: Sci Rep. 2026 Jul 23;16:23097. doi: 10.1038/s41598-026-62522-2 (PMC13396338; doi:10.1038/s41598-026-62522-2)
Supplement: Supplementary file 1 — Supplementary Material 1 [file 41598_2026_62522_MOESM1_ESM.pdf]

Supplementary Information to Article "Marine heatwaves in the Northeast Pacific intensify landfalling atmospheric rivers on the west coast of North America"

Christoph Renkl[1,2*], Hyodae Seo[2,3] and Arthur J. Miller[4]

[1*]University of Bonn, Bonn, Germany.
[2]Woods Hole Oceanographic Institution, Woods Hole, MA, USA.
[3] University of Hawai'i, Mānoa, Honolulu, HI, USA.
[4]Scripps Institution of Oceanography, University of California, San Diego, La Jolla, CA, USA.

*Corresponding author(s). E-mail(s): christoph.renkl@uni-bonn.de;

This PDF file includes:

- S1 SCOAR Northeast Pacific Configuration
  - Figure S1
  - Table S1
- S2 Experimental Design and Model Evaluation
  - Figures S2 & S3
- S3 Temporal Variation and Changes in AR-Related Variables
  - Figure S4
- S4 Latent Heat Flux Decomposition
  - Figure S5

S1 SCOAR Northeast Pacific Configuration

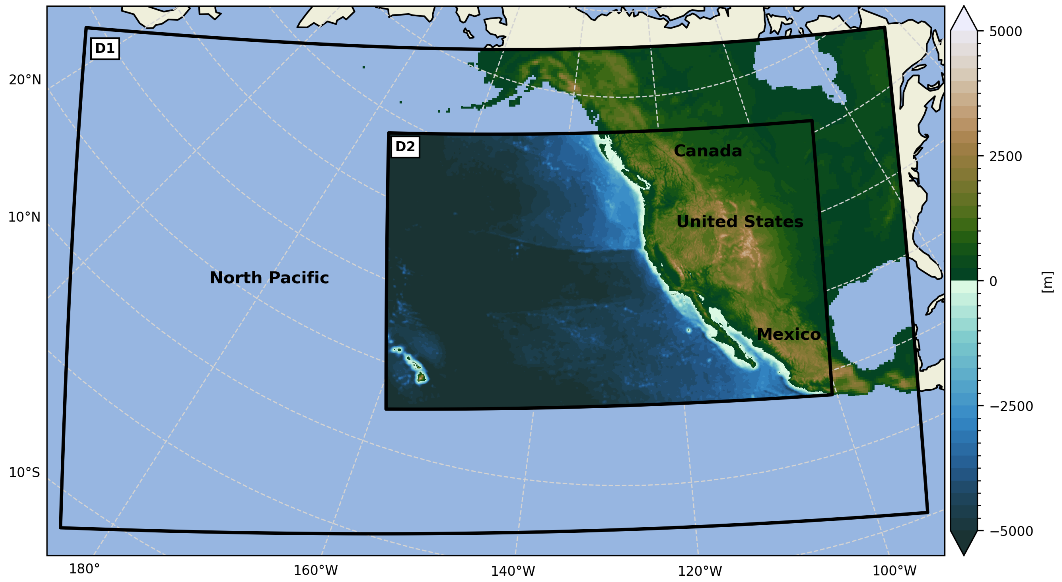

Fig. S1 Model domains and topography of the SCOAR configuration of the northeast Pacific. The atmosphere-only outer domain D1 (30 km grid spacing) is used to downscale the ERA5 reanalysis and provide lateral boundary conditions for the inner domain D2. In domain D2 (7.5 km grid spacing), WRF and ROMS are coupled via the COARE bulk parameterization and share the same land–sea mask. This map was generated using the Python package Cartopy version 0.25 (https://github.com/SciTools/cartopy).

Table S1 Summary of model physics schemes applied in this study.

Model	Physics	Scheme/Parameterization	Namelist Parameter
WRF	Cumulus parameterization[1]	Multi-Scale Kain-Fritsch (Zheng et al. 2016; Glotfelty et al. 2019)	`cu_physics = 11`
	Cloud microphysics	WRF Single-Moment 3-class (WSM3; Hong et al. 2004)	`mp_physics = 3`
	Radiation	New Rapid Radiative Transfer Model (RRTMG; Iacono et al. 2008)	`ra_[lw,sw]_physics = 4`
	Planetary boundary layer physics	Mellor-Yamada Nakanishi and Niino Level 2.5 (MYNN2; Nakanishi and Niino 2006, 2009; Olson et al. 2019)	`bl_pbl_physics = 5`
	Surface physics	Nakanishi and Niino PBL's Surface Layer Scheme (MYNN; Olson et al. 2019)	`sf_sfclay_physics = 5`
ROMS	Horizontal tracer advection	Third-Order Upstream-Biased (Shchepetkin and McWilliams 1998, 2005)	`Hadvection = U3`
	Vertical tracer advection	Fourth-Order Centered (Shchepetkin and McWilliams 2005)	`Hadvection = C4`
	Free surface boundary condition	Chapman (Chapman 1985)	`LBC(isFsur) = Cha`
	2D momentum boundary condition	Flather (Flather 1994)	`LBC(is[U,V]bar) = Fla`
	3D momentum boundary condition	Mixed Radiation–Nudging (Marchesiello et al. 2001)	`LBC(is[U,V]vel) = RadNud`
	Tracer boundary condition	Mixed Radiation–Nudging (Marchesiello et al. 2001)	`LBC(isTvar) = RadNud`
	Mixing TKE boundary condition	Gradient	`LBC(isMtke) = Gra`
	Horizontal mixing of tracers	Harmonic operator	`TNU2 = 10.0`
	Horizontal mixing of momentum	Harmonic operator	`VISC2 = 10.0`
	Vertical mixing	k-ϵ scheme, Generic Length Scale (GLS) formulation (Umlauf and Burchard 2003, 2005)	
	Stability function	Canuto-A (Canuto et al. 2001, 2002)	

[1]Only applied in the outer domain D1.

S2 Experimental Design and Model Evaluation

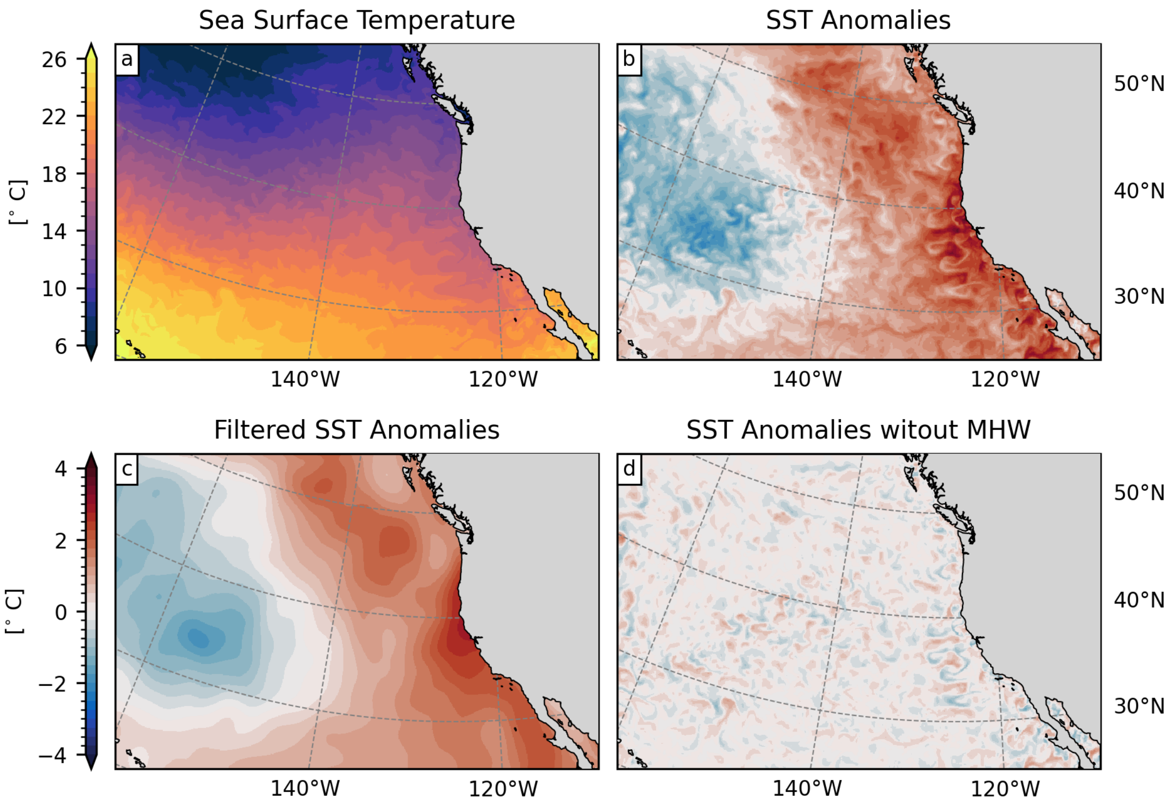

Fig. S2 Isolation of large-scale marine heatwave (MHW) from initial model conditions. (a) Predicted sea surface temperature (SST) at 00 UTC on 1 December, 2014 after the one-month coupled spin-up simulation. (b) SST anomalies at 00 UTC on 1 December, 2014 with respect to 10-year ocean-only daily climatology (2005–2014). (c) Large-scale MHW calculated by applying a locally estimated scatterplot smoothing (LOESS) smoother (Cleveland 1979; Schlax and Chelton 1992; Schlax et al. 2001), with half-width (effective cutoff) length scale of 5° to SST anomalies shown in panel (b). The same filtering is applied to temperature anomalies at each level of the ocean model component. (d) Resulting SST anomalies associated with meso-scale eddies after subtracting the large-scale MHW shown in panel (c) from the initial conditions. See Methods for details.

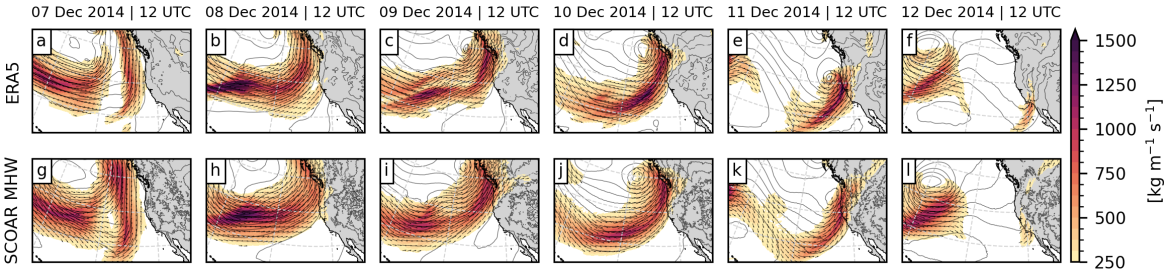

Fig. S3 Landfalling atmospheric rivers (ARs) during the period 7–12 December 2014 in ERA5 (top) and ensemble mean predicted by the coupled MHW simulation (bottom). Daily snapshots of vertically integrated water vapor transport (IVT, $\mathrm{kg\,m^{-1}\,s^{-1}}$; vectors at every 6th and 20th grid point, respectively, and shading, only values $> 250\,\mathrm{kg\,m^{-1}\,s^{-1}}$ are shown) and sea level pressure (SLP, hPa; contours in increments of 5 hPa).

S3 Temporal Variation and Changes in AR-Related Variables

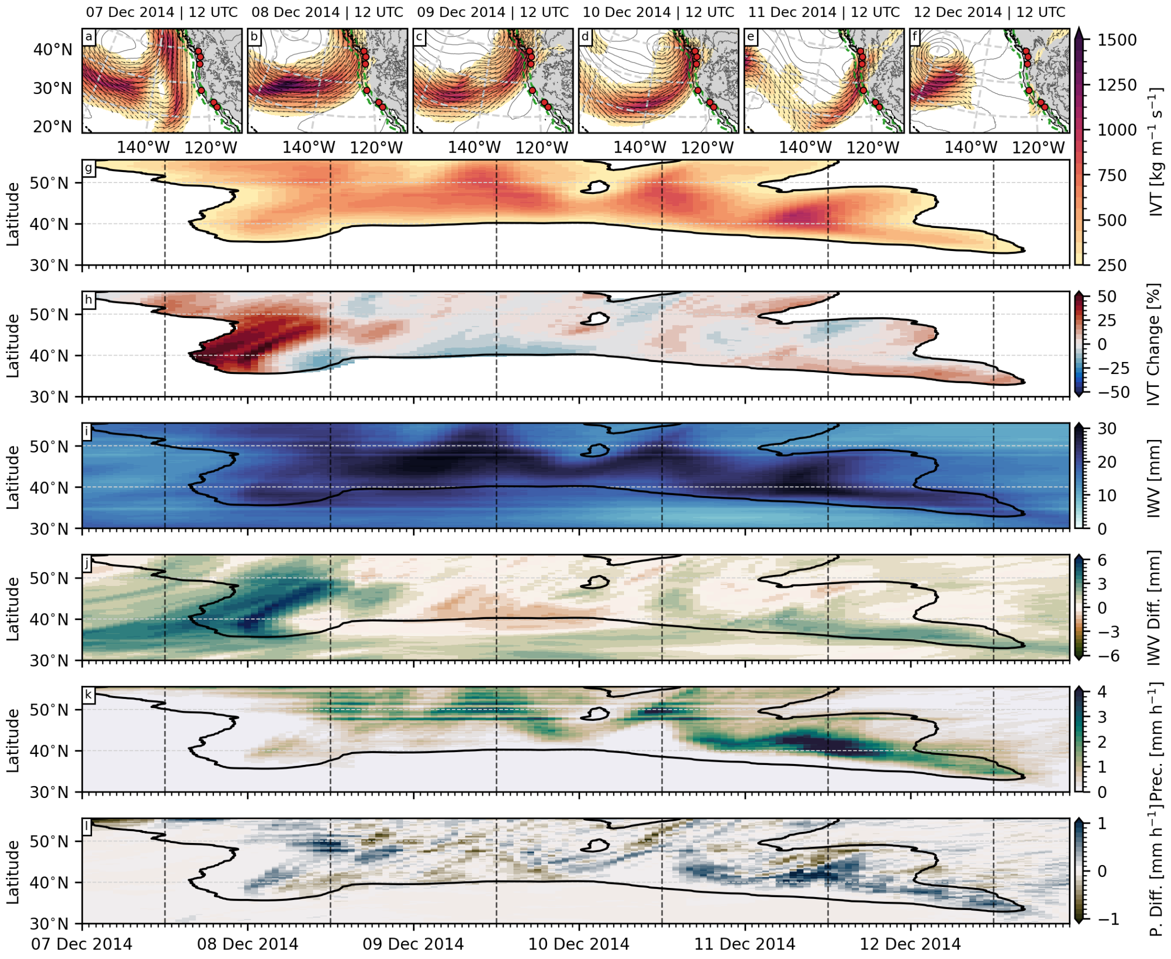

Fig. S4 Temporal variation and changes in variables related to landfalling atmospheric rivers (ARs) during the period 7–12 December 2014 predicted by the coupled model. (a)–(f) Daily snapshots of ensemble mean vertically integrated water vapor transport (IVT, $\mathrm{kg\,m^{-1}\,s^{-1}}$; vectors at every 20th model grid point and shading, only values $> 250\,\mathrm{kg\,m^{-1}\,s^{-1}}$ are shown) and sea level pressure (SLP, hPa; contours in increments of 5 hPa) based on the MHW simulation. Green dashed lines delineate the area ±50 km from the coast and red dots mark major cities. Hovmöller diagrams of (g) ensemble mean IVT (MHW simulation), (h) percentage change of ensemble mean IVT relative to the noMHW simulation, (i) ensemble mean IWV (MHW simulation), (j) differences in ensemble mean IWV (MHW − noMHW), (k) ensemble mean hourly precipitation rate (MHW simulation), (l) differences in ensemble mean hourly precipitation rate (MHW − noMHW). In the Hovmöller diagrams in panels g–l, all variables have been zonally averaged over the area ±50 km from the coast. Black contour marks IVT magnitude $>250\,\mathrm{kg\,m^{-1}\,s^{-1}}$ in the MHW simulation. Vertical dashed lines indicate the time at which the snapshots in panels a–f are taken. Panels a–g are the same as in Figure 2 of the main text and are repeated here for context.

S4 Latent Heat Flux Decomposition

The latent heat flux in the COARE framework (Fairall et al. 1996, 2003; Edson et al. 2013) is defined by the bulk formula (e.g., Cronin et al. 2019)

$$Q_\mathrm{l} = \rho_\mathrm{a} L_\mathrm{v} C_\mathrm{e} U (q_\mathrm{s} - q), \tag{1}$$

where ρ_a is the air density, L_v is the latent heat of evaporation, C_E is transfer coefficient, U is the wind speed relative to the ocean surface, q_s is the saturation-specific humidity, and q_a is the near-surface specific humidity. Positive values point upward, representing heat loss from the ocean to the atmosphere.

Differences in latent heat flux between the MHW and noMHW simulation can be approximated by the following linearized formulation (e.g., Cayan 1992)

$$\Delta Q_\mathrm{l} \approx \rho_\mathrm{a} L_\mathrm{v} C_\mathrm{e} \Delta U (q_\mathrm{s} - q_\mathrm{a}) + \rho_\mathrm{a} L_\mathrm{v} C_\mathrm{e} U \Delta[(q_\mathrm{s} - q)], \tag{2}$$

where

$$\Delta(\cdot) = (\cdot)_\mathrm{MHW} - (\cdot)_\mathrm{noMHW}. \tag{3}$$

This allows the differences to be separated into thermodynamic changes in the vertical humidity gradient $q_\mathrm{s} - q$ and dynamic changes in wind speed.

Figure S5 shows the differences in time-averaged ensemble latent heat flux due to large-scale SST anomalies as presented in the main text (Fig. 4e) and the contributions by thermodynamic and dynamic changes, respectively, estimated using (2). Here, U has been approximated by the 10 m wind speed, and $\rho_\mathrm{a} = 1.225\,\mathrm{kg\,m^{-3}}$, $L_\mathrm{v} = 2.5 \times 10^6\,\mathrm{J\,kg^{-1}}$, and $C_\mathrm{e} = 1.5 \times 10^{-3}$ are taken to be constant. This illustrates that the mean differences in latent heat flux between the two model simulations are predominantly due thermodynamic changes in the vertical humidity gradient (Fig. S5b). Since q_s is a function of SST, the MHW raises the saturation-specific humidity, thereby promoting enhanced evaporation and latent heat flux from the ocean.

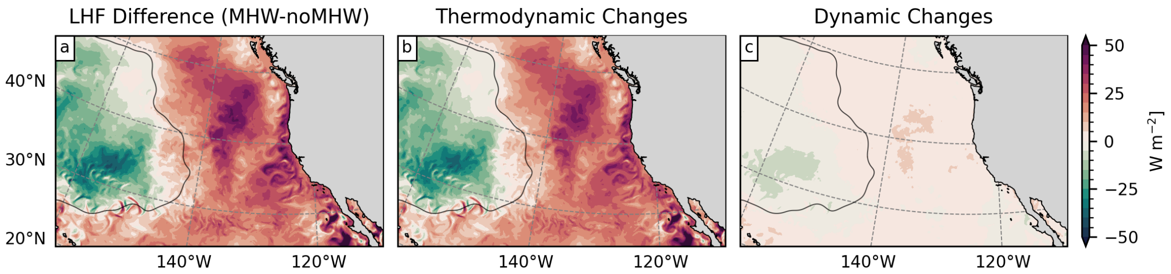

Fig. S5 Changes in time-averaged ensemble mean latent heat flux under MHW conditions derived from a linearized formulation of the bulk formula. (a) Differences (MHW − noMHW) in upward latent heat flux. This is the same as Fig. 4e. Contribution to changes in mean latent heat flux by (b) thermodynamic changes due to the vertical humidity gradient at the air–sea interface and (c) changes in wind speed at 10 m above the surface.

References

Cayan, D.R.: Latent and Sensible Heat Flux Anomalies over the Northern Oceans: The Connection to Monthly Atmospheric Circulation. Journal of Climate **5**(4), 354–369 (1992) 26197190

Cronin, M.F., Gentemann, C.L., Edson, J., Ueki, I., Bourassa, M., Brown, S., Clayson, C.A., Fairall, C.W., Farrar, J.T., Gille, S.T., Gulev, S., Josey, S.A., Kato, S., Katsumata, M., Kent, E., Krug, M., Minnett, P.J., Parfitt, R., Pinker, R.T., Stackhouse, P.W., Swart, S., Tomita, H., Vandemark, D., Weller, A.R., Yoneyama, K., Yu, L., Zhang, D.: Air-Sea Fluxes With a Focus on Heat and Momentum. Frontiers in Marine Science **6** (2019)

Chapman, D.C.: Numerical Treatment of Cross-Shelf Open Boundaries in a Barotropic Coastal Ocean Model. Journal of Physical Oceanography **15**(8), 1060–1075 (1985) https://doi.org/10.1175/1520-0485(1985)015⟨1060:NTOCSO⟩2.0.CO;2 . Chap. Journal of Physical Oceanography

Canuto, V.M., Howard, A.M., Cheng, Y., Dubovikov, M.S.: Ocean Turbulence. Part I: One-Point Closure Model—Momentum and Heat Vertical Diffusivities. Journal of Physical Oceanography **31**(6), 1413–1426 (2001) https://doi.org/10.1175/1520-0485(2001)031⟨1413:OTPIOP⟩2.0.CO;2

Canuto, V.M., Howard, A., Cheng, Y., Dubovikov, M.S.: Ocean Turbulence. Part II: Vertical Diffusivities of Momentum, Heat, Salt, Mass, and Passive Scalars. Journal of Physical Oceanography **32**(1), 240–264 (2002) https://doi.org/10.1175/1520-0485(2002)032⟨0240:OTPIVD⟩2.0.CO;2

Cleveland, W.S.: Robust Locally Weighted Regression and Smoothing Scatterplots. Journal of the American Statistical Association **74**(368), 829–836 (1979) https://doi.org/10.1080/01621459.1979.10481038

Edson, J.B., Jampana, V., Weller, R.A., Bigorre, S.P., Plueddemann, A.J., Fairall, C.W., Miller, S.D., Mahrt, L., Vickers, D., Hersbach, H.: On the Exchange of Momentum over the Open Ocean. Journal of Physical Oceanography **43**(8), 1589–1610 (2013) https://doi.org/10.1175/JPO-D-12-0173.1 . Chap. Journal of Physical Oceanography

Fairall, C.W., Bradley, E.F., Hare, J.E., Grachev, A.A., Edson, J.B.: Bulk Parameterization of Air–Sea Fluxes: Updates and Verification for the COARE Algorithm. Journal of Climate **16**(4), 571–591 (2003) https://doi.org/10.1175/1520-0442(2003)016⟨0571:BPOASF⟩2.0.CO;2

Fairall, C.W., Bradley, E.F., Rogers, D.P., Edson, J.B., Young, G.S.: Bulk parameterization of air-sea fluxes for Tropical Ocean-Global Atmosphere Coupled-Ocean Atmosphere Response Experiment. Journal of Geophysical Research: Oceans **101**(C2), 3747–3764 (1996) https://doi.org/10.1029/95JC03205

Flather, R.A.: A Storm Surge Prediction Model for the Northern Bay of Bengal with Application to the Cyclone Disaster in April 1991. Journal of Physical Oceanography **24**(1), 172–190 (1994) https://doi.org/10.1175/1520-0485(1994)024⟨0172:ASSPMF⟩2.0.CO;2

Glotfelty, T., Alapaty, K., He, J., Hawbecker, P., Song, X., Zhang, G.: The Weather Research and Forecasting Model with Aerosol–Cloud Interactions (WRF-ACI): Development, Evaluation, and Initial Application. Monthly Weather Review **147**(5), 1491–1511 (2019) https://doi.org/10.1175/MWR-D-18-0267.1 . Chap. Monthly Weather Review

Hong, S.-Y., Dudhia, J., Chen, S.-H.: A Revised Approach to Ice Microphysical Processes for the Bulk Parameterization of Clouds and Precipitation. Monthly Weather Review **132**(1), 103–120 (2004) https://doi.org/10.1175/1520-0493(2004)132⟨0103:ARATIM⟩2.0.CO;2 . Chap. Monthly Weather Review

Iacono, M.J., Delamere, J.S., Mlawer, E.J., Shephard, M.W., Clough, S.A., Collins, W.D.: Radiative forcing by long-lived greenhouse gases: Calculations with the AER radiative transfer models. Journal of Geophysical Research: Atmospheres **113**(D13) (2008) https://doi.org/10.1029/2008JD009944

Marchesiello, P., McWilliams, J.C., Shchepetkin, A.: Open boundary conditions for long-term integration of regional oceanic models. Ocean Modelling **3**(1-2), 1–20 (2001) https://doi.org/10.1016/S1463-5003(00)00013-5

Nakanishi, M., Niino, H.: An Improved Mellor–Yamada Level-3 Model: Its Numerical Stability and Application to a Regional Prediction of Advection Fog. Boundary-Layer Meteorology **119**(2), 397–407 (2006) https://doi.org/10.1007/s10546-005-9030-8

Nakanishi, M., Niino, H.: Development of an Improved Turbulence Closure Model for the Atmospheric Boundary Layer. Journal of the Meteorological Society of Japan. Ser. II **87**(5), 895–912 (2009) https://doi.org/10.2151/jmsj.87.895

Olson, J.B., Kenyon, J.S., Angevine, W.A., Brown, J.M., Pagowski, M., Sušelj, K.: A Description of the MYNN-EDMF Scheme and the Coupling to Other Components in WRF–ARW. Technical Report NOAA Technical Memorandum OAR GSD-61, Earth System Research Laboratory, Boulder CO, USA (2019)

Schlax, M.G., Chelton, D.B.: Frequency Domain Diagnostics for Linear Smoothers. Journal of the American Statistical Association **87**(420), 1070–1081 (1992) https://doi.org/10.1080/01621459.1992.10476262

Schlax, M.G., Chelton, D.B., Freilich, M.H.: Sampling Errors in Wind Fields Constructed from Single and Tandem Scatterometer Datasets. Journal of Atmospheric and Oceanic Technology **18**(6), 1014–1036 (2001) https://doi.org/10.1175/1520-0426(2001)018⟨1014:SEIWFC⟩2.0.CO;2 . Chap. Journal of Atmospheric and Oceanic Technology

Shchepetkin, A.F., McWilliams, J.C.: Quasi-Monotone Advection Schemes Based on Explicit Locally Adaptive Dissipation. Monthly Weather Review **126**(6), 1541–1580 (1998) https://doi.org/10.1175/1520-0493(1998)126⟨1541:QMASBO⟩2.0.CO;2 . Chap. Monthly Weather Review

Shchepetkin, A.F., McWilliams, J.C.: The regional oceanic modeling system (ROMS): A split-explicit, free-surface, topography-following-coordinate oceanic model. Ocean Modelling **9**(4), 347–404 (2005) https://doi.org/10.1016/j.ocemod.2004.08.002

Umlauf, L., Burchard, H.: A generic length-scale equation for geophysical turbulence models. Journal of Marine Research **61**(2), 235–265 (2003) https://doi.org/10.1357/002224003322005087

Umlauf, L., Burchard, H.: Second-order turbulence closure models for geophysical boundary layers. A review of recent work. Continental Shelf Research **25**(7-8), 795–827 (2005) https://doi.org/10.1016/J.CSR.2004.08.004

Zheng, Y., Alapaty, K., Herwehe, J.A., Genio, A.D.D., Niyogi, D.: Improving High-Resolution Weather Forecasts Using the Weather Research and Forecasting (WRF) Model with an Updated Kain–Fritsch Scheme. Monthly Weather Review **144**(3), 833–860 (2016) https://doi.org/10.1175/MWR-D-15-0005.1 . Chap. Monthly Weather Review
